# Using CNN Features to Better Understand What Makes Visual Artworks Special

**DOI:** 10.3389/fpsyg.2017.00830

**Published:** 2017-05-23

**Authors:** Anselm Brachmann, Erhardt Barth, Christoph Redies

**Affiliations:** ^1^Experimental Aesthetics Group, Institute of Anatomy, School of Medicine, Jena University Hospital, University of JenaJena, Germany; ^2^Institute of Neuro- and Bioinformatics, University of LübeckLübeck, Germany

**Keywords:** experimental aesthetics, visual art, statistical image properties, self-similarity, richness, CNN feature responses, aesthetic perception

## Abstract

One of the goal of computational aesthetics is to understand what is special about visual artworks. By analyzing image statistics, contemporary methods in computer vision enable researchers to identify properties that distinguish artworks from other (non-art) types of images. Such knowledge will eventually allow inferences with regard to the possible neural mechanisms that underlie aesthetic perception in the human visual system. In the present study, we define measures that capture variances of features of a well-established Convolutional Neural Network (CNN), which was trained on millions of images to recognize objects. Using an image dataset that represents traditional Western, Islamic and Chinese art, as well as various types of non-art images, we show that we need only two variance measures to distinguish between the artworks and non-art images with a high classification accuracy of 93.0%. Results for the first variance measure imply that, in the artworks, the subregions of an image tend to be filled with pictorial elements, to which many diverse CNN features respond (*richness* of feature responses). Results for the second measure imply that this diversity is tied to a relatively large *variability* of the responses of individual CNN feature across the subregions of an image. We hypothesize that this combination of richness and variability of CNN feature responses is one of properties that makes traditional visual artworks special. We discuss the possible neural underpinnings of this perceptual quality of artworks and propose to study the same quality also in other types of aesthetic stimuli, such as music and literature.

## 1. Introduction

In experimental aesthetics, researchers try to understand the perceptual basis of aesthetic experience and its neural correlates in the human brain. On the one hand, in neuroaesthetics, modern brain imaging methods have been used to investigate which brain regions are involved in the processing of aesthetic stimuli (Pearce et al., 2016). On the other hand, by analyzing image statistics, contemporary methods in computer vision enable researchers to identify properties in images of artworks that distinguish them from other (non-art) types of images (Graham and Redies, 2010). Eventually, these two areas of experimental aesthetics will have to be joined to reach a deeper understanding of where and how the physical structure of esthetically pleasing images is processed in the human brain, and how such brain responses can be modeled.

In the present work, we focus on the second goal of experimental aesthetics, which is to understand what is special about the structure of aesthetic stimuli. Computational aesthetics, a subfield of computer vision, has contributed various tools and algorithms to tackle this question. For a long time, work in this field was dominated by features that reflected common *a priori* knowledge about aesthetic principles. Such hand-crafted features were used mainly to predict aesthetic ratings of photographs (Datta et al., 2006; Dong et al., 2015).

For example, Datta et al. (2006) collected 3,581 photographs together with their aesthetic ratings from the online community *Photo.net*. They defined a set of multiple features, which were then used with an SVM classifier to predict high vs. low ratings for their set of photographs with an accuracy of 70.12% using a set of 15 different features. The feature set included low-level features like color saturation and hue, as well as more abstract features, such as the adherence to the Rule of Thirds, which is popular among photographers. Ke et al. (2006) proposed a method to distinguish high-quality images and low-quality snapshots, using features like the spatial distribution of edges, contrast and hue, as well as blur. Following this idea, several researchers have proposed other measures in order to predict aesthetic quality ratings (Luo and Tang, 2008; Wong and Low, 2009; Nishiyama et al., 2011).

A different approach was taken by Marchesotti et al. (2011), who did not design features by hand, but adopted generic image descriptors to predict the quality of photographs. The authors used multiple SIFT (Lowe, 2004) descriptors to encode gradient information, as well as a color descriptor for capturing color; their method outperformed their predecessors significantly (classification accuray of 89.90% compared to 75.85% with the features proposed by Datta et al., 2006, and to 76.53% with the features proposed by Ke et al., 2006).

Visual artworks, such as paintings and artistic drawings, have been studied to a lesser extent (Graham and Redies, 2010). For example, Li and Chen (2009) applied the idea of extracting multiple features for quality assessment to paintings and proposed a set of 40 different features in order to capture art concepts like harmony and balance.

There have also been various attempts to characterize visual artworks by focusing on single or very few image properties. For example, Taylor (2002) investigated the drip paintings of the American abstract painter Jackson Pollock and found that he painted fractals similar to those found in nature. Moreover, large subsets of visual artworks and natural scenes share a similar scale-invariant power spectrum (Graham and Field, 2007; Redies et al., 2007). Redies et al. (2012) investigated the distribution of luminance edges in artworks and found that it is rather uniform and about as self-similar as that of natural scenes. Together, these studies indicated that large subsets of visual artworks share specific statistical properties with natural scenes (Graham and Redies, 2010). Because the human visual system is adapted to process images of our natural habitat efficiently, it has been suggested (Taylor, 2002; Redies, 2007) that aesthetic quality of visual stimuli relates to the adaptation of the human visual system to natural scenes (Simoncelli and Olshausen, 2001).

More recently, we analyzed the layout of edge orientations in images of diverse sets of artworks from various cultural backgrounds by comparing pairwise the orientation of each edge in an image with the orientations of all other edges in the same image (Redies et al., 2017). Results revealed that the artworks are characterized by a lack of correlations between edge orientations across an image. A promising research question in computational aesthetics therefore seems to be how much *variability* there is in the pictorial elements of images in general and how this variability is distributed across images of artworks. A more intuitive insight into this question is that visual artworks display a harmonious arrangement of their pictorial elements, which has also been called “good Gestalt” (Arnheim, 1954) or “visual rightness” (Locher et al., 1999). We hypothesize that this type of aesthetic quality can manifest itself in the distribution of variability in colors, edges and other visual features across an artwork. In the present work, we investigate this variability by using features from Convolutional Neural Networks (CNNs).

Due to recent progress in object recognition, CNNs have gained a huge popularity (Krizhevsky et al., 2012; Simonyan and Zisserman, 2014; He et al., 2015), although the basic idea underlying CNNs was already proposed more than thirty years ago (Fukushima, 1980; LeCun and Bengio, 1995). Part of the present success can be explained by advancements in computing technology and the availability of a huge amount of data for training (LeCun et al., 2015). CNNs learn a hierarchy of filters, which are applied to an input image in order to recognize image content. While lower-level features are very general and resemble those found in human early vision (Yosinski et al., 2014), higher-level features learn rich semantic representations (Donahue et al., 2014). The training of a CNN is done in a supervised manner using backpropagation, an algorithm that compares the current output of the model to a target output. This procedure allows propagating the calculated error back through the network and changing the parameters of each filter according to its contribution.

With the growing popularity of CNNs, these models have also been applied to image rating tasks (Dong et al., 2015; Lu et al., 2015) and proved to be well-suited to predict aesthetic quality as well. For example, CNNs were used to distinguish colored paintings of Western provenance from non-artistic images by using an SVM classifier (Denzler et al., 2016). The downside of deploying deep models and end-to-end feature learning, however, is that a model's performance does not always allow drawing conclusions on how the model's features bring about this performance. In other words, while it is possible to predict whether an image will be considered to be aesthetic or an artwork, it is not easy to understand why this might be the case. In another line of studies, CNNs have been used to transfer the visual characteristics of particular artistic styles to photographs (Gatys et al., 2015).

Typically, CNNs are trained on datasets containing thousands of categories and millions of example images to recognize objects or scenes. The learned features were shown to be suitable for generic recognition tasks (Donahue et al., 2014; Hertel et al., 2015). Here, we exploit this property and apply the CNN features to artworks and, for comparison, to other types of images. However, because CNNs are trained to recognize objects or scenes, the training approach may not be feasible to study the nature of artworks because artworks can depict almost any object or scene. We therefore decided to ask how artworks are processed by CNN filters, rather than to use CNNs to detect specific image content in artworks. We focus our study on features at lower levels of CNNs because that is where generic (i.e., content-independent) responses are found (Yosinski et al., 2014). Moreover, higher levels may not be suitable because the content displayed in artworks may be different from the images that the CNNs were trained on and therefore provide indifferent activations by these stimuli. Similar approaches using low-level generic CNN features have recently been proposed for detection of the left/right symmetry in images (Brachmann and Redies, 2016), for quality assessment of image reproductions (Amirshahi et al., 2016), and for measuring self-similarity in images (Brachmann and Redies, in press).

With regards to the variability of visual features in artworks, there has been no *comprehensive* and *systematic* analysis by the computational approach to date. Rather, as stated above, individual aspects of image structure, such as color distribution, edge orientation and the spatial frequency spectrum, were analyzed separately without assessing overall similarities in their variance or distribution. The present study represents a first attempt to overcome this problem.

To carry out a *comprehensive* study, CNN features are particularly useful because they resemble the human visual system in how they encode input images. Notably, CNN features from lower layers reflect several primordial neural mechanisms of human vision (Wurtz and Kandel, 2000). In parallel, they process information on luminance gradients and their orientation, color and spatial frequency, and thus allow us to link the CNN features to several different processes underlying human visual perception.

To carry out a *systematic* study, we calculate three types of variance (total variance, variance across features, and variance across image patches) for a wide range of spatial scales. Results for artworks are compared to various other categories of man-made stimuli and natural patterns and scenes. Based on only two of the variance measures, we succeed in defining a feature space, in which colored artworks can be discriminated with an accuracy of 93% from other types of colored images, in particular photographs of man-made and natural patterns and scenes. Our findings hold for colored traditional artworks from diverse provenances (Western, Islamic and East Asian cultures), suggesting that the here-defined features space can be applied across cultures.

In conclusion, there have been two seemingly divergent approaches in computational aesthetics. On the one hand, the usage of multiple image features, including diverse CNN features, has allowed predicting the aesthetic quality of photographs and artworks with reasonable accuracy, but the interpretability of this success in terms of the underlying visual processes remains limited. On the other hand, particular visual properties have been identified in visual artworks and were related to the visual processing of natural patterns and scenes; however, these properties are not unique to artworks. In the present study, we combine these two approaches. By using multiple lower-level CNN filters, we study particular statistical measures, i.e., the variability of the CNN features, to ask whether there is a particular pattern of features that distinguishes colored artworks from diverse types of real-world photographs.

## 2. Methods

### 2.1. Variance of CNN features

**Figure 1:**
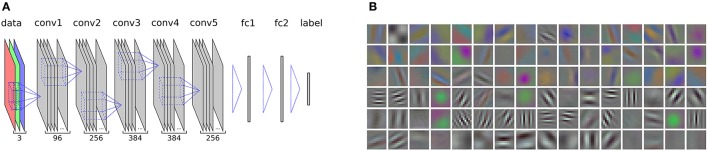
**(A)** A schematic representation of the AlexNet CNN, which we use in our experiments. The layers that are output of a filtering operation are shown in gray (*conv*: convolutional layers, *fc*: fully-connected layers). Filter operations between the layers are represented by dashed blue lines. The numbers at the bottom indicate the total number of different filter maps at each layer. A hierarchic, consecutive filtering of the input image, which is decomposed into its red, green, and blue channel (see *data* layer), allows to extract features that are well suited to recognize objects in an image and to assign a class *label*. In our experiments, we dropped the fully-connected layers above the last convolutional layer (*conv5*) in order to be able to rescale the network to a higher resolution of the convolutional layers. The normalization layers and pooling layers of the model are not shown. For a detailed description of the model, see Krizhevsky et al. (2012). **(B)** A visualization of the 96 filters on the first convolutional layer, which detect oriented luminance edges at different frequencies as well as opponent color gradients.

In order to discriminate visual artworks from other image categories, we use the features of the well-established AlexNet model, which was first introduced in Krizhevsky et al. (2012) and is provided in the CaffeNet Library (Jia et al., 2014). A schematic diagram of the model along with a visualization of the first-layer filters is shown in Figure 1. The original input dimension of the network is 227 × 227 pixels, which proved to be sufficient for object recognition. However, much detail of the original image is lost when it is scaled down to such low resolution. In order to preserve the amount of detail that may be required for a full aesthetic evaluation, we drop the fully-connected layers and rescale the input dimension to 512 × 512 pixels, while keeping the filter sizes and parameters as defined in the original model. A similar upscaling was carried out by Gatys et al. (2015) who showed that information on image style and content is preserved after upscaling.

We measure variances of features as follows: For a given convolutional layer *l*, we divide each filter response map into *n* × *n* equally sized subregions and record the highest responses for each subregion and every filter on that layer, i. e., we perform a max-pooling operation over the response maps. This provides us with a max-pooling map *M*_*l,n*_, which has three dimensions: two positional parameters defining the position of the regions, over which the max-pooling is performed, and a third dimension for each filter, with *k* being the total number of filters on the respective layer *l*.

After a normalization of the histograms of each subregion, which assured that all filter responses of every subregion summed are equal to one, we measure three different variances:

1. The total variance over *a*ll entries of *a*ll histograms:
  (1) $$\begin{array}{r} p_{a}\left(M_{l,n}\right)=\text{var}\left(M_{l,n}\right) \end{array}$$
2. The median variance of the histograms of the *n* × *n* subregions of the *g*rid:
  (2) $$\begin{array}{r} v_{g}\left(M_{l,n}\right)=\left(\text{var}\left(M_{l,n}\left(x,y,i\right)|i\in 1..k\right)|x,y\in 1..n\right) \end{array}$$
  (3) $$\begin{array}{r} p_{g}\left(M_{l,n}\right)=\text{median}\left(v_{g}\left(M_{l,n}\right)\right) \end{array}$$
3. The median over the variance of each *f* ilter:
  (4) $$\begin{array}{r} v_{f}\left(M_{l,n}\right)=\left(\text{var}\left(M_{l,n}\left(x,y,i\right)|x,y\in 1..n\right)|i\in 1..k\right) \end{array}$$
  (5) $$\begin{array}{r} p_{f}\left(M_{l,n}\right)=\text{median}\left(v_{f}\left(M_{l,n}\right)\right) \end{array}$$

For convenience, we will denote *p*_*V*_(*M*_*l,S*_) as *P*_*V*_(*S*) for a given convolutional layer *l*, so that *P*_*a*_(12) would stand for *p*_*a*_(*M*_*l*,12_). We calculate each of the above variance measures for different numbers of subimages *n* ranging from 2 to 30 in steps of 2. This provides us with 45 values in total, which describe the overall variance of features of a given image. The choice of the above variance measures was motivated by our aim to capture abstract aesthetic concepts like “visual rightness” (Locher et al., 1999) in terms of the distribution of colors and edges distributed across the image. With respect to the hypothesis formulated in the Introduction, we are interested in the question of which subset of these values is the most discriminating for artworks compared to other categories of images. To answer this question, we test subsets of different sizes from the pool of variances for their discrimination power and used them as input features for a Support Vector Machine (SVM) with a Radial Basis Function (RBF) kernel that is trained to assign the labels *art* and *non-art* for a given image. SVMs separate classes by maximizing the margin between a hyperplane and the nearest training examples, called the support vectors (for an introduction to SVMs, see Bishop, 2006). To avoid overfitting, we carry out 5-fold cross-validation and take the mean of all five test sets as our final classification value for a given feature set.

### 2.2. Image datasets

In our study, a total of nine different image categories are compared. Three categories comprise artworks and six categories contain non-art images. One of the artwork categories, the JenAesthetics dataset, consists of a total of 1629 images of traditional oil paintings of Western provenance (Amirshahi et al., 2013). The images from this dataset cover different art periods like Renaissance, Baroque, Romanticism, Realism, Impressionism and Expressionism, ranging from the fifteenth to the twentieth century. Moreover, images are annotated according to subject matters (e. g., portrait, landscape, still life).

In order to expand our analysis to artworks from other cultures, we also include a dataset of illustrations from traditional Islamic illuminated books and miniatures (251 images) and traditional colored brush paintings from China (210 images) in our study (Redies et al., 2017). All images were downloaded from the Wikimedia Commons database (https://commons.wikimedia.org). A particular effort was made to select images with well-preserved colors and sufficiently high resolution. A list of all images with the individual links to the Wikimedia database can be obtained from the authors.

**Figure 2:**
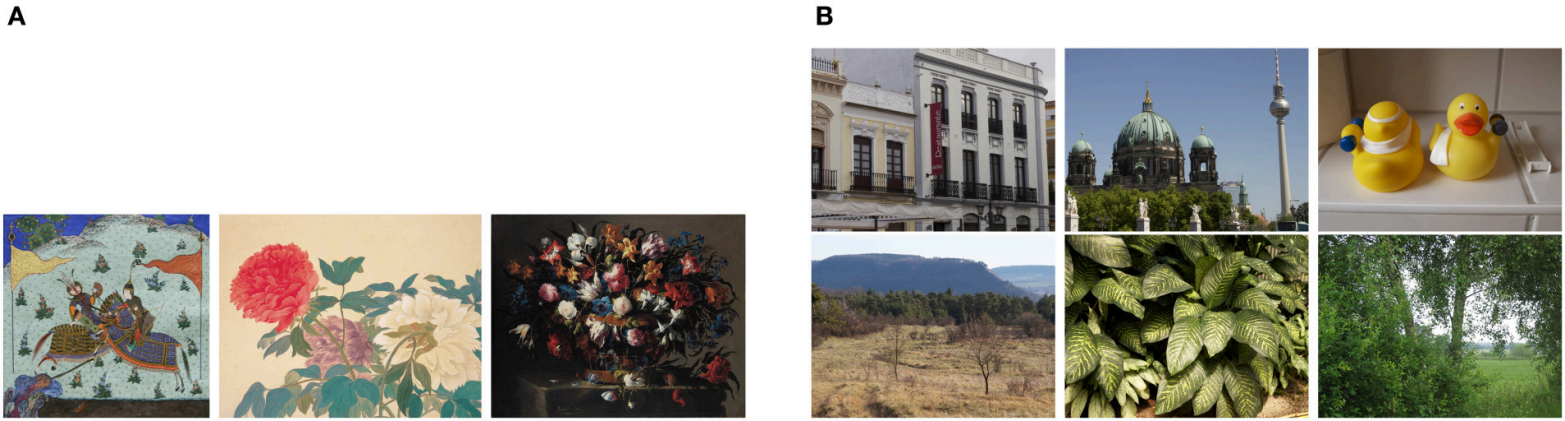
**Example images of the datasets used in present study**. **(A)** Examples of artworks (from left to right: Islamic art, Chinese art, and Western art from the JenAesthetics dataset). **(B)** Examples of non-art images from the categories Buildings, Urban Scenes, Objects, Large Vistas, Plant Patterns and Vegetation. See Table 2 for values of *P*_*f*_(12) and *P*_*a*_(22) for each image.

To compare results for artworks with images of other man-made scenes and objects, the non-artistic categories include photographs of buildings (511 images), urban scenes (219 images) and simple objects (207 images). Moreover, previous studies demonstrated that some statistical properties are shared between artworks and natural scenes and images, in particular with large-vista natural scenes (see Section 2). As a particular challenge in the classification task, we therefore include photographs of large-vista natural scenes (473 images), of plant patterns (331 images) and of vegetation (458 images) in our analysis. All non-art images were taken with a Canon EOS 500D camera by one of the authors (Redies et al., 2012; Braun et al., 2013). The photographs were taken and processed in RAW or TIF format to avoid compression artifacts. Example images are shown in Figure 2. In total, we thus use 4,289 images (2,090 images as examples for artworks and 2199 images of non-artistic content). This distribution is roughly balanced and entails a best-guessing probability of 0.513 when always choosing the same class. We also report results from a set of photographs of lichen growth patterns (244 images), which are extremely self-similar and complex (Redies et al., 2012). Finally, to address the question whether the analysis might possibly be biased by camera artifacts, we also included the first 2,090 images of the ImageNet test set in the analysis (Russakovsky et al., 2015).

## 3. Results

**Figure 3:**
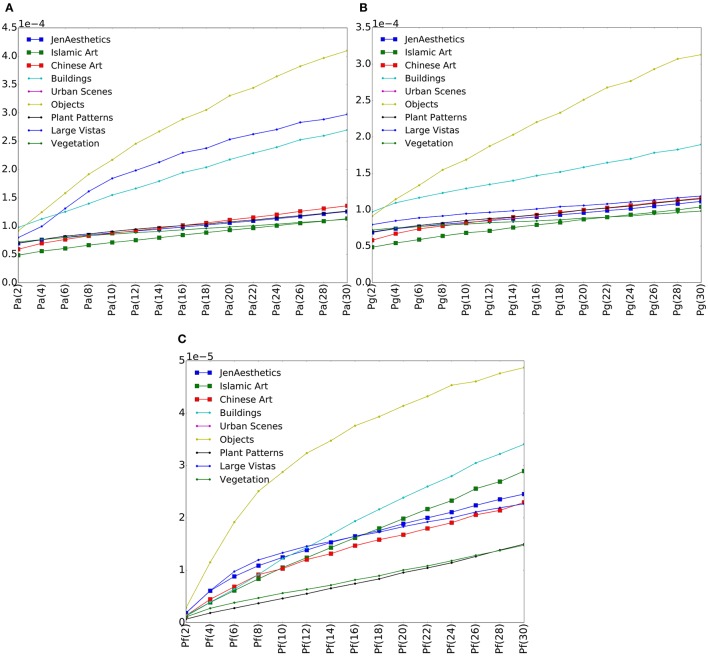
**Plots of median variances ***P***_***a***_ (A)**, *P*_*g*_ **(B)**, and *P*_*f*_ **(C)** for each category on convolutional layer 1. Values for artworks (larger symbols) are similar, but differ from those of the other image categories (smaller symbols).

We calculated the three variance measures proposed in Section 2 for all images in our dataset. Figure 3 shows different variance plots calculated on convolutional layer *conv1*, the first convolutional layer of the AlexNet model. All three measures increase in value with more patches and therefore smaller patch sizes, as more and more detail of an image is taken into account. For different image categories, different slopes are observed. Objects, for example, show a much steeper slope than most of the other categories for all three values. The categories containing art, namely images from the JenAesthetics dataset (Western art) as well as Islamic art and Chinese art, show similar slopes for all measures. For *P*_*a*_, the curves for art are similar to those for Plant Patterns and Vegetation, whereas for *P*_*f*_, they are similar to Large Vistas for smaller patch sizes, while values for Plant Patterns and Vegetation are lower.

In order to test whether the different variance measures can be used to discriminate art images vs. non-art images, we trained an SVM classifier, as described in Section 2. We apply 5-fold cross-validation and define the average accuracy on the test samples of all five models as the classification accuracy of the respective subset. The higher this accuracy is, the more specific the chosen feature set is for artworks. We test sets that contain one feature, two features or three features in combination. Each set defines a space, in which image categories form clusters.

**Figure 4:**
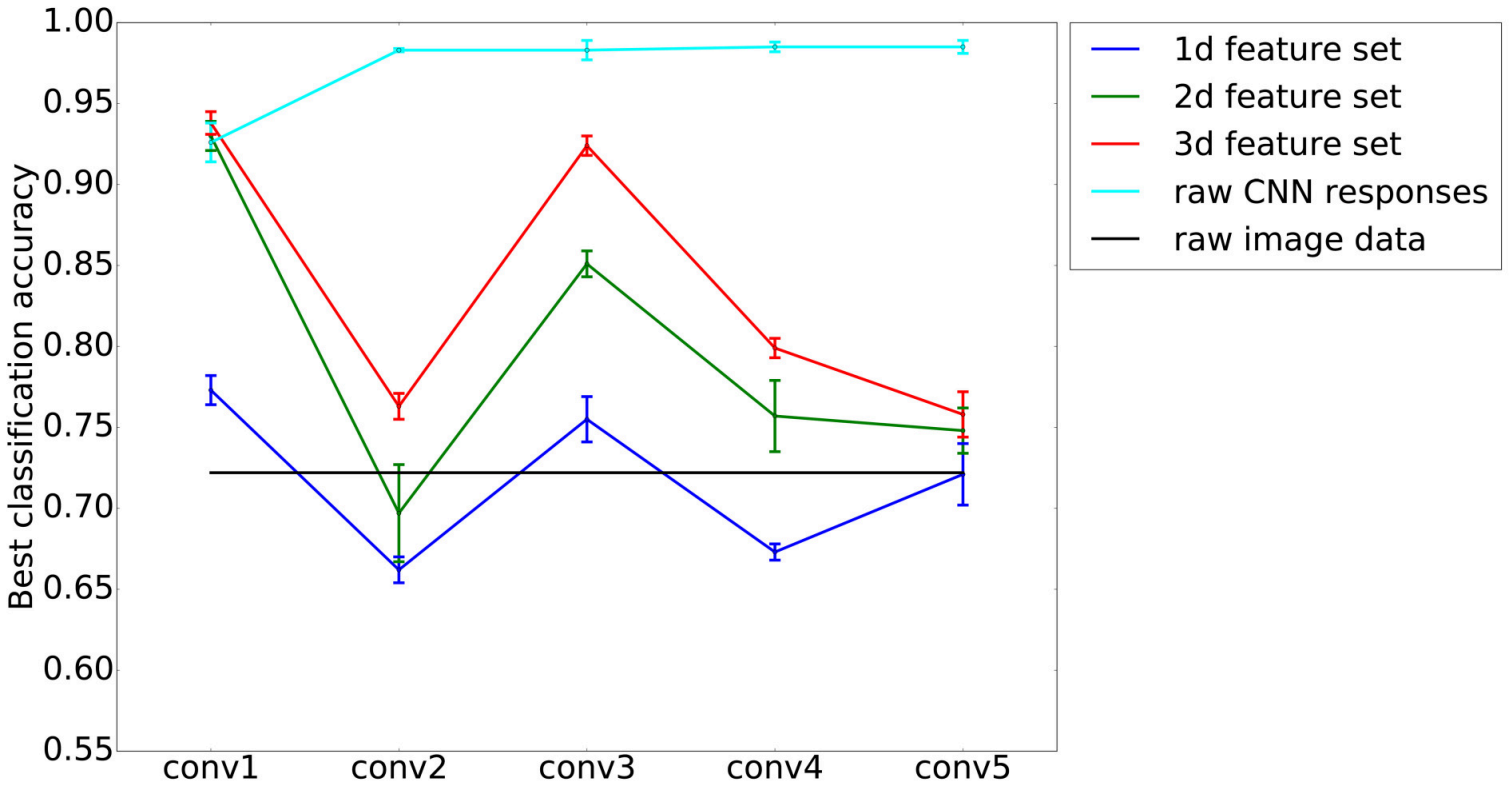
**Best classification performances for different layers, using sets of features that define spaces of different dimensions (1d–3d feature sets; ***blue, green*** and ***red***, respectively)**. As expected, a higher dimensionality yields a better classification accuracy. Interestingly, on the first convolutional layer *(conv1)* of the AlexNet model, a two-dimensional set of features performs almost as well as a three-dimensional one. The values represent means and the error bars *SD*s from a 5-fold cross-validation experiment. Two baselines as provided for comparison. First, a linear SVM on the raw CNN responses of each respective layer (*cyan*), and second, a linear SVM on the raw image data (*black*).

Figure 4 shows the maximum classification performance when discriminating traditional art vs. non-art images for different set sizes and on different layers. As expected, the classification performance decreases with less features in a set and correspondingly lower dimensions of the feature space. The best classification performances are obtained with three features on the first convolutional layer, yielding a maximum accuracy of 0.938 ± 0.007 *SD*, compared to a correct guessing probability of 0.513. Notably, the second best performance is obtained with a features set containing only two features: Combining features *P*_*f*_(12) and *P*_*a*_(22), an SVM classifier is able to discriminate art from non-art images with an accuracy 0.930 ± 0.009 *SD*. In the remainder of this article, we will focus our analysis on features obtained on the first convolutional layer. For this analysis, classification results are listed in Table 1 for the different categories of art images.

**Table 1:** **Mean classification scores (±***SD***) for art images vs. non-art images, according to different categories of artworks**.

**Category**	**Accuracy**	**Precision**	**Recall**
Western art (*m* = 1629)	0.939 ± 0.008	0.931 ± 0.005	0.926 ± 0.017
Chinese art (*m* = 210)	0.851 ± 0.012	0.354 ± 0.018	0.852 ± 0.038
Islamic art (*m* = 251)	0.957 ± 0.015	0.726 ± 0.083	0.964 ± 0.008
All combined (*m* = 2,090)	0.930 ± 0.004	0.942 ± 0.009	0.913 ± 0.010

*While for Western art (JenAesthetics dataset) and Islamic art, the classification score is above 90%, it drops to 85% for Chinese art. Here, the mean precision is low, due to an overall lower richness (i.e., higher values of P_a_(22)). Results are from a 5-fold cross-validation experiment. m, number of images in the dataset*.

For comparison, we provide two different baselines. First, we tested the classification of a linear SVM on the raw red, green, and blue pixel values of a downscaled version (227 × 227 pixels, as the input of the unmodified AlexNet model) of the images in our experiment. This downscaling was necessary because of computing power limitations and reduced the input dimensions of the resulting features space to a total of 3·227^2^ = 154, 587 dimensions. Here, we obtained a mean classification performance of 0.722 ± 0.014 *SD* (see black line in Figure 4). Second, we tested the classification performances of a linear SVM on the raw filter maps that were obtained from processing it with the AlexNet model. Here, dimensions are even higher: For example on the first convolutional layer, the input dimension amounts to a total of 290,400 (96 filters at a resolution of 55 × 55 pixels). Results for the baselines are shown in Figure 4.

**Figure 5:**
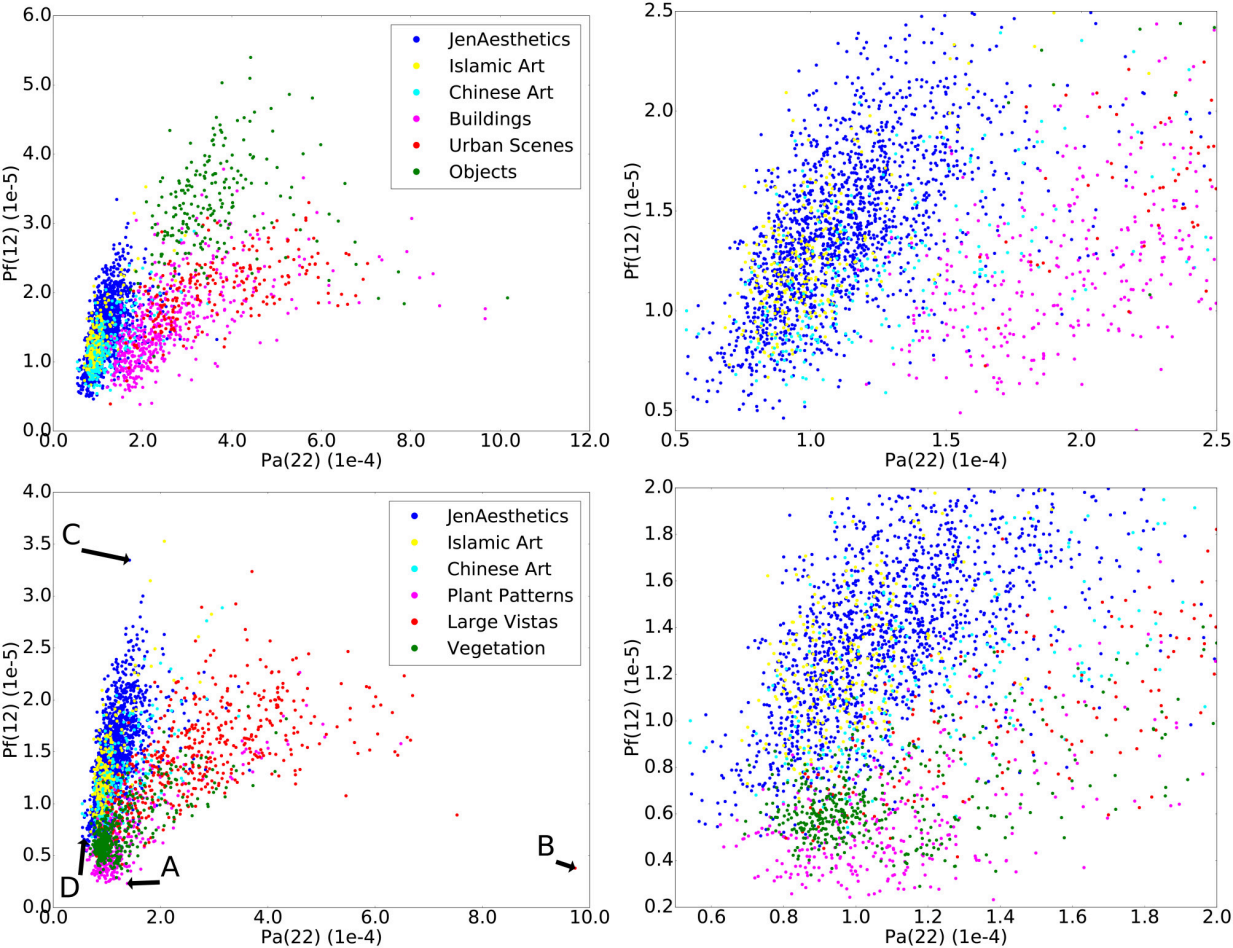
**The figure visualizes the space defined by the two features ***P***_***a***_(22) and ***P***_***f***_(12), which were calculated on the first convolutional layer**. All four panels show art categories (blue, yellow, and cyan), which form a distinct cluster. On the top row, art categories are compared to categories that show man-made scenes, and on the bottom row, art is compared to natural scenes and patterns. The left-hand side of the figure shows the entire space, while the right-hand side magnifies the central region of interest of the space. Categories overlap only partly, which is reflected in the high classification accuracy that we obtain in our experiment. The example images shown in Figure 6 are marked by uppercase letters A, B, C, and D, the respective values are given in Table 2.

**Figure 6:**
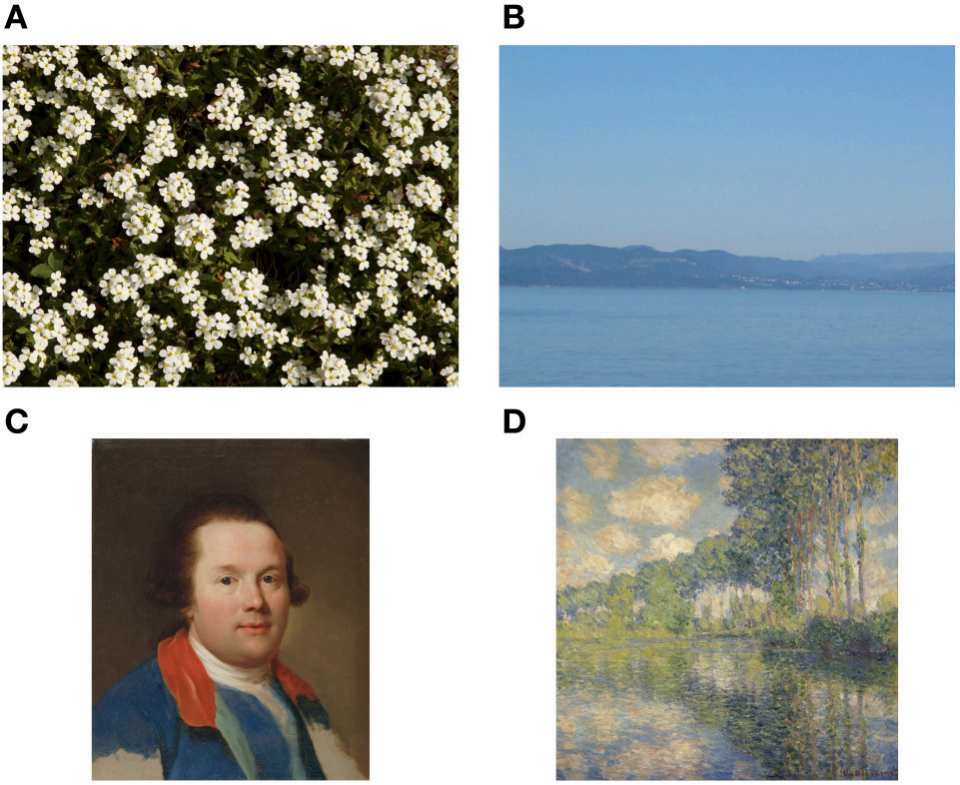
**Extreme cases of images that serve to illustrate the space that best separates art images from non-art images (Figure **5**)**. **(A,B)** show non-art images, while **(C,D)** show artworks. The image of the plant pattern in **(A)** is rich in structure (low *P*_*a*_(22)) but is highly self-similar with little variability across the image (low *P*_*f*_(12)). The large vista scene in **(B)** represents a highly homogeneous image that lacks structure like edges or different colors (high *P*_*a*_(22)). The painting in **(C)** (A. R. Mengs, 1769) is atypical in that it contains large, relatively homogeneous regions (high *P*_*f*_(12)), similar to objects. The painting in **(D)** (Monet, 1891) has a very rich structure (low *P*_*a*_(22)) but it is not as self-similar (higher *P*_*f*_(12)) as most images of plant patterns, vegetation or lichen growth patterns. See Table 2 for values of *P*_*f*_(12) and *P*_*a*_(22).

Figure 5 visualizes the space defined by the two features and makes intuitively accessible why the classification can be done with such high accuracy. Figure 6 shows extreme cases of images embedded in this space. The variance values for each image are listed in Table 2. In this feature space, different image categories form clusters, which overlap to some extent, but overall, art images are separated from non-art images. To put this separation into perspective and rule out a possible artifactual bias in our dataset of photographs, we also calculated classification performances for comparing art with the equivalent amount of images from the ImageNet (Russakovsky et al., 2015) test set of photographs. Here, features *P*_*f*_(10) and *P*_*a*_(16) result in a top classification accuracy of 0.861 when combined. With a set of three features (*P*_*a*_(10), *P*_*f*_(18), and *P*_*f*_(30)), we reach a top classification accuracy of 0.871 for the first convolutional layer.

**Table 2:** **Values of features ***P***_***a***_(22) and ***P***_***f***_(12) for the images shown in Figures **2**, **6**, **8**, **9****.

**Figures**	**Image**	***P*_*a*_(22) (1*e*−4)**	***P*_*f*_(12) (1*e*−5)**
2A	Islamic art by Bashdan Qara	1.05	1.48
2A	Chinese art by Yun Shouping	2.40	2.21
2A	Western painting by J. DeArellano	0.93	1.26
2B	Buildings	2.04	1.28
2B	Urban scene	7.03	2.42
2B	Objects	3.78	5.03
2B	Large vista scene	2.98	1.87
2B	Plant pattern	1.33	1.06
2B	Vegetation	0.94	0.57
6A	Plant pattern (flowers)	1.38	0.23
6B	Large vista scene (lake)	9.73	0.24
6C	Western painting by A. Mengs	1.42	3.35
6D	Western painting by C. Monet	0.57	0.66
8A	Plant pattern	1.24	1.35
8B	Western painting by M. Chase	3.65	1.32
8C	Large vista scene	1.02	1.02
8D	Western painting by P. Cezanne	0.98	0.75
9A	Plant pattern (moss)	1.02	1.07
9A	Plant pattern (flowers)	1.24	1.35
9A	Large vista scene (waterfall)	1.08	1.16
9A	Large vista scene	1.41	1.78
9A	Vegetation (poppy flowers)	0.99	1.10
9A	Vegetation (pond)	1.03	1.20
9A	Vegetation (forest)	0.94	1.21
9A	Large vista scene (river)	1.05	1.39
9A	Large vista scene (rocks)	0.93	1.21
9B	Chinese art by Lang Shining	2.47	1.31
9B	Western painting by H. Avercamp	2.55	1.33
9B	Chinese art by Xu Yang	0.98	0.54
9B	Western painting by E. Manet	0.98	0.52
9B	Western painting by C. Monet	1.37	0.74
9B	Western painting by H. LeSidaner	1.45	0.78
9B	Western painting by C. Monet	0.90	0.46
9B	Western painting by C. Hassam	1.22	0.64
9B	Western painting by E. Boudin	1.93	0.98

Figure 4 shows that, from the first to the second convolutional layer, the maximum accuracy of classification drops for all sizes of feature sets, but rises again for the third convolutional layer. This means that, while variances of features are distinctive for art on the first and the third convolutional layer, they are less specific for artworks compared to other image categories on the second and fourth layer.

**Figure 7:**
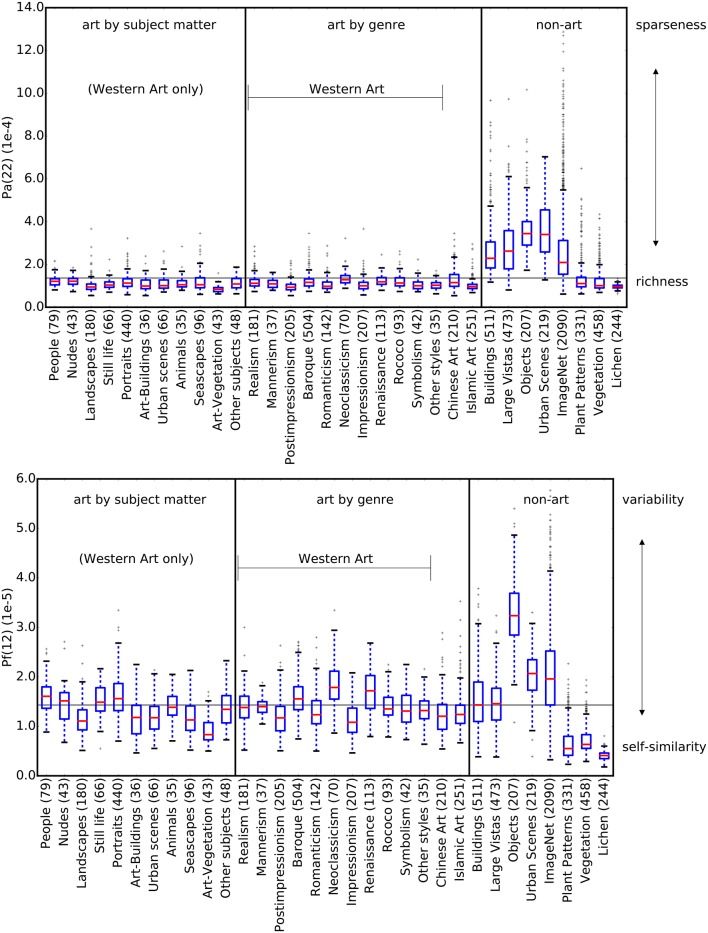
**The plots show the two features ***P***_***a***_(22) (top)** and *P*_*f*_(12) **(bottom)**. The horizontal line repesents the median of all values in both plots. Art images are grouped according to subject matter (left section) and art genre (middle section). For *P*_*a*_(22), all subsets of artworks show a similar distribution, regardless of genre and subject matter, which are similar to the non-art categories Plant Patterns, Vegetation and Lichen. The distributions for feature *P*_*f*_(12) among categories of art are more diverse compared to *P*_*a*_(22), but nevertheless distinctly different from Plant Patterns, Vegetation and Lichen. The numbers in parentheses indicate the number of images in each dataset.

Figure 7 shows values for *P*_*a*_(22) and *P*_*f*_(12) for artworks grouped by genre and subject matter, compared to non-artworks. For *P*_*a*_, artworks show similar mean values and distributions, independent of subject matter or art genre (Figure 7 top). This similarity holds true also for images of other cultural provenances (Islamic and Chinese artworks). Feature *P*_*f*_(12), which is plotted in Figure 7 bottom, shows more variable distributions for subject matter and art genres. Neoclassicism, for example, shows higher values than other genres. Nevertheless, this feature contributes to the high classification accuracy because all genres and subjects have higher values than the non-art categories Plant Patterns and Vegetation, which are similar to art with regard to feature *P*_*a*_ (Figure 7 top).

## 4. Discussion

The aim of the present study was to identify characteristics of traditional artworks by focusing on the variances of CNN features. In the present study, we restrict our analysis to color images because the filters that we use were trained on color images and, as a result, developed features for color processing. We chose to analyze variances (for a definition, see Section 2) because we speculated that such measures could help us to capture the distribution of color and luminance edges across an image, which seems to be an important characteristic of artworks (see Introduction). Our results show that features derived from different layers are suitable to discriminate art from non-art images (see Figure 4 for a comparison), with lower-layer features having the highest discriminatory power.

We compared our classification performances to two different baselines. First, we find that, for the first convolutional layer, sets of one, two, or three features are higher than for a classification done on the raw pixel channels (*data* in Figure 1). Second, we determined the classification performances on the raw filter responses on different layers of the AlexNet model and found that the performance is always above 90% (Figure 4). Keeping in mind that, in these experiments, the dimensionality is orders of magnitudes higher than in our original experiment (290,400 dimensions vs. 1, 2, or 3 dimensions), our results can be considered a success in terms of distilling relevant information from this high-dimensional stream of data. Furthermore, we gain interpretability, which is nearly impossible in the high-dimensional case.

### 4.1. Interpretation of the variances *P*_*a*_ and *P*_*f*_

The feature set *P*_*a*_(22) and *P*_*f*_(12) is the two-dimensional set that is best suited for classification of traditional art vs. non-art images in our study; it yields an overall classification rate of 0.930. While the interpretation of responses from higher-layer feature remains problematic and is a matter of ongoing research (Zeiler and Fergus, 2014; Yosinski et al., 2015), first-layer features, such as those analyzed in the present work, are more accessible to analysis and interpretation. By interpreting what these features measure, we can distill two main characteristics of artworks.

First, the mean overall variance of all filter responses in all patches, as measured by feature *P*_*a*_, is significantly lower for the artworks than for Buildings, Large Vistas, Objects and Urban Scenes, but comparable to that of Plant Patterns and Vegetation (Figure 7). We interpret *P*_*a*_ as a measure for the degree of *sparseness* in filter responses in an image and of their distribution in all image subregions. The value is high if, for any given patch, only one or a few of filters respond, that is, if the histogram of the patch contains only one or a few bins that reach high values, while most other bins show low values. High *P*_*a*_ values are observed in images that are homogeneous in many of their subregions, for example, in images that show a large blue sky or large objects with uniformly colored surfaces (for example, see Figure 6B). Conversely, low values, as observed for artworks, Plant Patterns, Vegetation and lichen growth patterns, indicate that a large number of filters respond to a similar degree in each patch. As a consequence, the variance between filter responses decreases. Theoretically, *P*_*a*_ approaches zero if all filters in all patches are activated to the same degree. We therefore interpret low *P*_*a*_ values as an indication of the *richness* of filter responses across an image.

Second, our interpretation of *P*_*f*_ is that it measures the inverse of *self-similarity* in an image because it represents the median variance of the responses of each filter across all subregions. We here call this property *variability*. When specific features in an image are very similar across all subregions, self-similarity is high and the median variance (or variability) is low. A low *P*_*f*_ thus indicates that a low number of subregions have filter responses that diverge from the other subregions. To confirm this relation, we also calculated self-similarity with the method proposed by Brachmann and Redies (in press) for all images used in our experiment. In this alternative method, self-similarity measures the degree to which features repeat themselves over the entire image. Specifically, image features are measured by performing a max-pooling operation over the entire image, which we refer to as the *ground level*. Then, a feature vector is build that holds the maximum responses of all features. Subsequently, as described for the present method, the image is split into 64 equally sized subregions, for which the max-pooling operation is carried out as well. The resulting feature vectors are then compared to the ground-level responses in order to measure the similarity between the entire image and its parts. Comparing feature *P*_*f*_(12) and the alternative self-similarity value for the first layer, we found a Spearman's correlation coefficient of −0.81, which corroborates the notion that *P*_*f*_(12) represents the inverse of self-similarity (i.e., variability).

Interestingly, images of artworks, plant patterns, vegetation and lichen growth patterns share similarly low *P*_*a*_(22) values (Figure 7), but they differ in their *P*_*f*_(12) values. Plant patterns, vegetation and lichen growth patterns are both rich in filter responses (low *P*_*a*_(22)) and show low variability (low *P*_*f*_(12); i.e., high self-similarity). Perceptually, this lack of variability results in the subjective impression that similar pictorial elements (e.g., blossoms, leaves or branches) are rather evenly distributed across the image (see Figure 6A). Although artworks show a similar richness of filter responses (low *P*_*f*_(12)), their filter responses are much more variable across the image (higher *P*_*f*_(12)). Images of objects, buildings and urban scenes also result in high variability of filter responses (high *P*_*f*_(12)), but their filter responses are more sparse overall (high *P*_*a*_(22) values).

**Figure 8:**
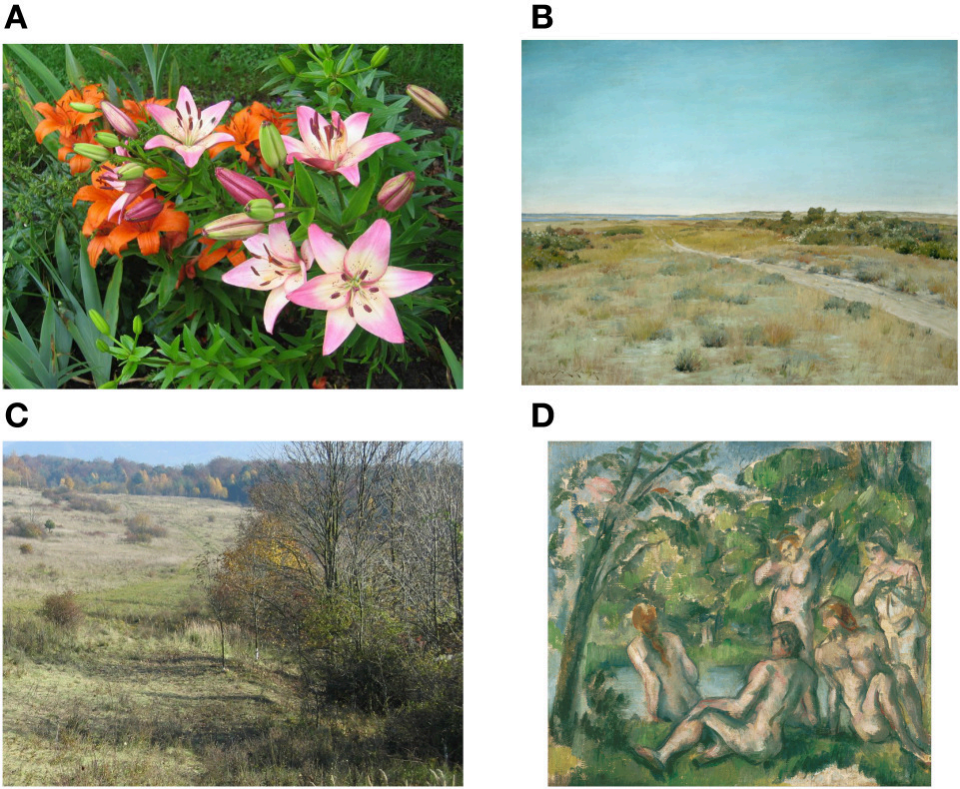
**Examples of non-art images misclassified as art (A,C)** and art images misclassified as non-art **(B,D)**. Both the photographs of flowers **(A)** and the large vista scene **(C)** have variances of features (edges and color) typical for artworks. The image “First Touch Of Autumn” by American painter W. M. Chase **(B)** is untypical for artworks in its homogeneity of the sky and the ground. The artwork “The Bathers” painted by P. Cezanne **(D)** is untypically self-similar for an artwork. See Table 2 for values of *P*_*f*_(12) and *P*_*a*_(22) for each image.

In conclusion, CNN filter responses are relatively rich and variable across the images of artworks. We speculate that the richness of filter responses across visual artworks corresponds to the subjective impression that most artworks contain a wealth of pictorial structure. In other words, artists tend to fill most parts (here defined by the grid we use for the calculation of the histograms) of their artworks with structure, such as luminance edges or different colors. Indeed, most of the artworks studied here display relatively few areas that are large, homogeneous or blank (i.e., areas of high *P*_*a*_; for an exception, see Figure 8B). This finding distinguishes artworks from all of the other categories in our experiment, except for photographs of plant patterns, vegetation and lichen growth patterns (Figure 7). We also found that the structural variability is high in artworks so that their structure does not become monotonous (as in most plant patterns and vegetation). Possibly, this variability—in combination with the richness of filter responses—might contribute to what has been called “good Gestalt” and “visual rightness” of artworks (see Introduction). Moreover, variability might sustain the observer's attention to the various details distributed over an artwork. Here, we call this model the *richness/variability model*.

**Figure 9:**
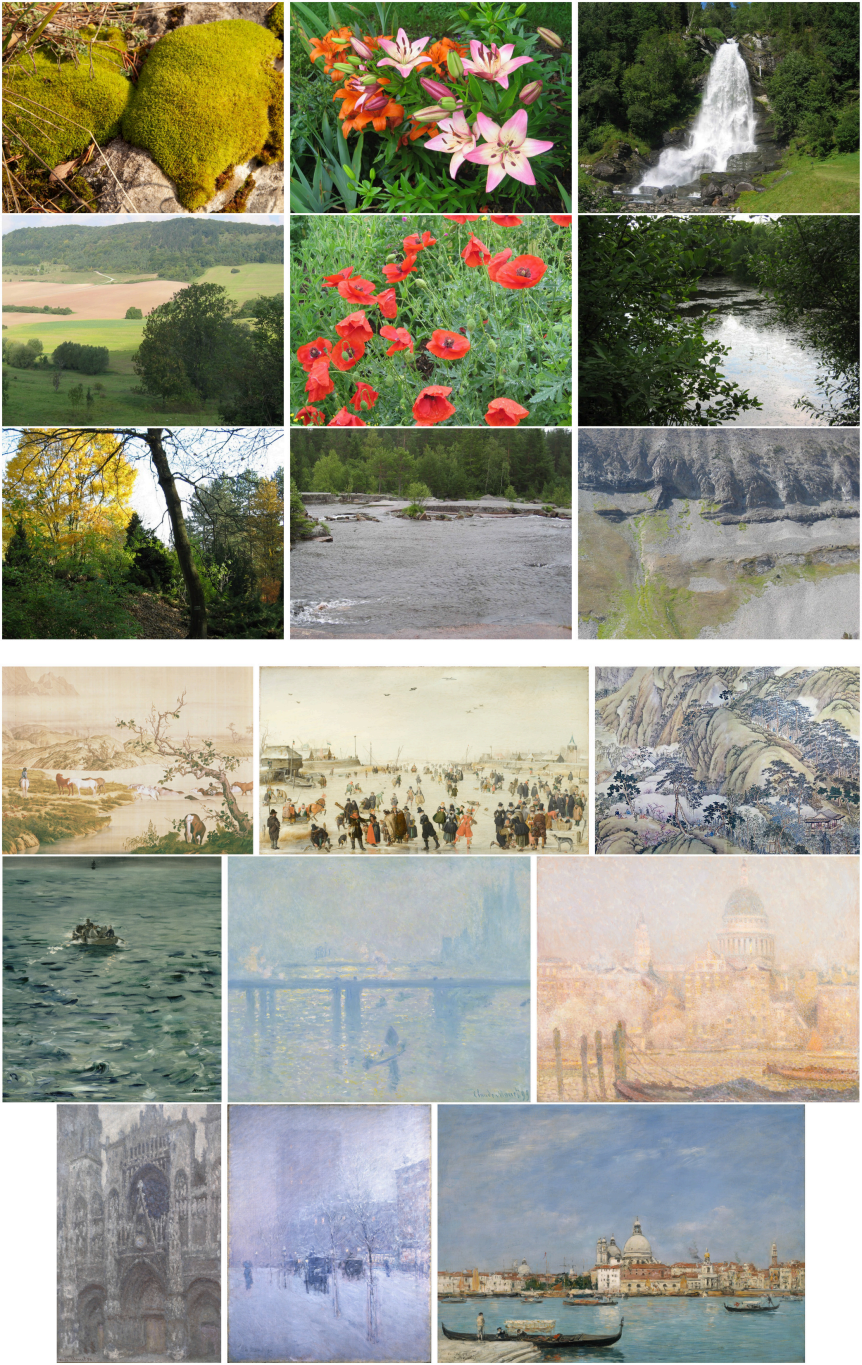
**The most strongly misclassified images, based on their distance to the decision boundary of the SVM classifier**. The top half of the figure shows non-art images misclassified as artworks, the bottom half shows artworks misclassified as non-art images. The *P*_*a*_(22) and *P*_*f*_(12) values of each image are listed in Table 2.

For an even better understanding of our model, Figure 8 shows examples of misclassified images. The variance values for each image are listed in Table 2. The left columns depicts non-art images classified as art, and the right column shows artworks misclassified as non-art images. While the images on the left side are easily recognized as regular photographs, the images on the right side are easily recognized as paintings. These examples indicate that the classification is not simply based on the recognition of painting technique, for example brush-stroke patterns. Rather, the distinction between artworks and non-art images relates to image features, such as the variance of colors and edges over subregions of the image (see the legend to Figure 8 for further details). The most strongly misclassified images are shown in Figure 9.

The present results can be compared to findings from two previous studies, which focused on the distribution of luminance edge orientations in large subsets of traditional artworks. First, anisotropy of luminance gradient orientations is low (Redies et al., 2012) and first-order entropy of edge orientations is high in the artworks (Redies et al., 2017). Both findings imply that the orientations of luminance gradients tend to be distributed uniformly across orientations in large subsets of artworks. This result is compatible with the present finding that filter responses, which include features that respond to luminance edges of various orientations, are rich in images of artworks. Second, by comparing edge orientations pairwise across artworks, we demonstrated that edge orientations tend to be independent of each other across artworks (Redies et al., 2017). At least for the CNN features that respond to luminance edges, we would therefore predict that the filter responses are not only variable but also distributed randomly across the artworks.

It should be stressed that the above findings are restricted to the sets of color images analyzed in the present study. For monochrome artworks, the color components of the CNN filters will not be as responsive and the model may require modifications. Moreover, the richness/variability model can be expected to fail for many (post-)modern and contemporary artworks. For example, the monochrome paintings by Yves Klein (e.g., *Untitled Blue Monochrome* [IKB 82], 1959) or the simple geometrical artworks by Kasimir Malevich (e.g., *Black Square*, 1915) are neither rich nor variable in their visual structure. In future studies, it will be of interest to investigate the differences between traditional art styles, which were analyzed in the present study, and (post-)modern artworks in more detail.

Last but not least, the richness/variability model is general enough to be applied to aesthetic stimuli in other sensory or cognitive modalities, such as music (Brattico et al., 2017) or literature. Prerequisite for such an application would be a set of physiologically plausible filters that can be used to extract perceptually meaningful structural information from the stimuli, and computer-based algorithms to calculate (i) the overall variance of all filter responses across the subparts of the stimuli (similar to *P*_*a*_ in Equation 1), and (ii) the mean variance of each filter response across the subparts (similar to *P*_*f*_ in Equation 5).

### 4.2. Possible underlying neural mechanisms

Converging evidence from neuroimaging studies suggests that multiple brain regions are involved in aesthetic experience. Examples are the orbitofrontal cortex, the cingulate cortex, the insula, the nucleus accumbens and the caudate nucleus (Brown et al., 2011; Cela-Conde et al., 2011; Vartanian and Skov, 2014). Some of these brain regions respond not only to visual artworks but also to aesthetic stimuli of other sensory and cognitive modalities, extending from the visual arts to music and even mathematics (Ishizu and Zeki, 2011, 2013; Zeki et al., 2014).

On the one hand, it has been argued that such brain responses provide a universal neural basis for aesthetic experience (Zeki, 2013). Indeed, a multiregional representation of aesthetic experience is suggested by the three pillars on which most current models of aesthetic experience are based: cognition, perception and emotion (Leder et al., 2004; Chatterjee and Vartanian, 2014). Moreover, there is a significant overlap between brain regions that are involved in aesthetic experience and neural mechanism of reward (Ishizu and Zeki, 2011), moral judgement (Zaidel and Nadal, 2011) and introspection (Vessel et al., 2013), all of which are mediated by specific networks in the human brain.

On the other hand, the present and previous results suggest that the physical structure of large subsets of visual artworks may follow relatively basic physical principles (see Introduction). It is therefore possible that basic mechanisms of aesthetic perception may already be implemented in lower cortical visual areas. However, most of the statistical image properties that have been associated with aesthetic stimuli to date reflect global statistical image properties that are likely to be based on long-distance interactions in the human visual system. For the primary visual cortex, very long-range interactions that extend beyond the surround of the classical receptive field (“association field”; Field et al., 1993) are not well established. We therefore speculated (Redies, 2015; Redies et al., 2017) that aesthetic perception may be mediated at visual cortical areas beyond the primary visual cortex, where contextual processing becomes more prominent, for example, in scene-sensitive brain regions.

Fundamental to visual aesthetics is that artworks can be created in many different styles and in almost any visual modality. Therefore, the various types of visual artworks are likely to stimulate visual channels differentially. Examples are monochrome graphic artworks (presumably addressing the luminance channel), Impressionist paintings (color channel), or artistic videos (movement channel). It is therefore conceivable that aesthetic experience is based on a neural mechanism that is implemented in most, if not all, visual channels (Redies, 2015), rather than by a mechanism that is found in only one of a few higher cortical brain regions. The detection of richness and variability of sensory information, which is the focus of the present work, might be such a general neural mechanism. The same (or a similar) mechanism might be implemented also in other sensory systems, the motor system or in cognitive neural circuits (see above). Consequently, it seems reasonable to postulate the existence of a general mechanism of aesthetic processing that is present in multiple sensory and cognitive channels, as proposed previously for the visual domain (“beauty-responsive mechanism”; Redies, 2015). If this mechanism is activated, it may then interact with neural circuits that process more specific emotional and cognitive aspects of aesthetic experience, including cultural, contextual or individual factors, which all contribute to aesthetic experience (Leder et al., 2004; Zeki, 2013; Chatterjee and Vartanian, 2014; Redies, 2015).

## 5. Conclusion

Motivated by a search to find statistical image properties that are special for artworks, we present a relatively simple algorithm that allows distinguishing artworks from other categories of images. In order to capture loosely defined concepts, such as visual rightness and good composition, we focused on variances of CNN features in our analysis. We chose to focus on filters that were obtained by training CNNs on millions of images because these features are good models of human vision for two reasons. First, the low-level CNN filters encode an image by using oriented luminance edges and color in form of opponent-color contrasts, similar to the human visual system. Second, higher CNN layers capture more abstract image content by combining low-layer features. Using a simple classification scheme, we identified two main properties in our analysis that, in combination, make artworks special. First, artworks tend to be filled with structure over the entire image *(richness)*, which separates them from almost all image categories in our analysis, except for some natural patterns. Second, artworks show more *variability* of the features across the image than the natural patterns, such as plant patterns and vegetation. Finding these systematic variances helps us to understand how aesthetic quality of artworks may be reflected in simple statistical properties. Many current efforts in computational aesthetics, particularly those that use large sets of image features to infer aesthetic ratings (Lu et al., 2015; Kao et al., 2016), lack this interpretability. While mimicking aesthetic judgment by training a deep network might provide useful insights, we here model the structure of artworks based solely on the activation of low-level features, which are known to work well for a variety of inputs. This approach might help to understand activation patterns and their relation to aesthetic perception and its neural underpinnings.

## Author contributions

AB and CR designed the study. AB wrote the code, carried out the experiments and analyzed the data. AB and CR wrote the manuscript. EB contributed to the conception of the study and helped draft the manuscript.

## Funding

This work was supported by institutional funds provided by the Institute of Anatomy, University of Jena School of Medicine.

### Conflict of interest statement

The authors declare that the research was conducted in the absence of any commercial or financial relationships that could be construed as a potential conflict of interest.
